# Supplementary material for: FlowMol3: flow matching for 3D de novo small-molecule generation
Source: Digit Discov. 2026 Apr 7;5(5):2052–66. doi: 10.1039/d5dd00363f (PMC13104864; doi:10.1039/d5dd00363f)
Supplement: DD-005-D5DD00363F-s001 [file DD-005-D5DD00363F-s001.pdf]

Supplementary Information (SI) for Digital Discovery.
This journal is © The Royal Society of Chemistry 2026

FlowMol3 Supplementary Information

Contents

S1 Additional Data on PoseBusters Validity 2

S2 Example Molecules 4

S3 The Coupling Distribution 4

S4 Training Details and Hyperparameters 5
- S4.1 Optimizer and Training Configuration 6
- S4.2 Model Architecture Hyperparameters 7
- S4.3 Flow Matching Hyperparameters 8

S5 Additional Techniques for DFM Sampling 9
- S5.1 Remasking via Stochasticity 9
- S5.2 Low-Temperature Sampling 10
- S5.3 Purity Sampling 10

S6 GVP with Cross Product 11

S7 Examples of Out-of-Distribution Ring Systems 11

S8 Individual Functional Group Frequencies 14

S9 Self-Conditioning Proportion Ablation 17

S10 Geometry Distortion Hyperparameter Ablation 18

S11 Loss Weight Sensitivity Analysis 21

S12 Fake Atom Hyperparameter Ablation 23

S13 Ring System Statistics **25**

S14 Speed–Quality Trade-off **26**

S15 On the Intractability of Quantifying Distribution Drift **28**
S15.1 Introduction and Motivation 28
S15.2 Background: The Flow Matching Framework 29
S15.2.1 Probability Paths and the Continuity Equation 29
S15.2.2 Marginal Vector Fields as Intractable Expectations . . 29
S15.3 Candidate Definitions of Drift 30
S15.3.1 Velocity Field Discrepancy 30
S15.3.2 Distributional Divergence 30
S15.3.3 Accumulated Trajectory Error 31
S15.3.4 Conditional Drift (Tractable but Uninformative) 31
S15.4 Fundamental Obstruction: The Marginalization Barrier 32
S15.5 What Can Be Measured: Proxy Metrics 32
S15.6 Connection to Self-Conditioning and Distortion 33
S15.7 Conclusion 33

S1 Additional Data on PoseBusters Validity

In the main paper we report the percent of molecules that are valid as determined by the PoseBusters suite[1] under the name "% PB-Valid". A model is considered PB-valid if it passes all of a series of tests. Here we present the pass-rate for each individual test run by PoseBusters; these results are shown in Table S1.

Table S1: Pass rates on individual PoseBusters tests with 95% confidence intervals

	FlowMol3	SemlaFlow	Megalodon	ADiT
mol pred loaded	100.0 ± 0.0	100.0 ± 0.0	100.0 ± 0.0	100.0 ± 0.0
sanitization	100.0 ± 0.0	95.4 ± 0.5	94.7 ± 0.2	99.9 ± 0.0
inchi convertible	100.0 ± 0.0	95.4 ± 0.5	94.7 ± 0.3	99.9 ± 0.0
all atoms connected	99.5 ± 0.2	97.3 ± 1.1	97.8 ± 0.5	94.6 ± 0.5
bond lengths	100.0 ± 0.0	99.6 ± 0.2	99.9 ± 0.1	96.0 ± 0.3
bond angles	100.0 ± 0.0	100.0 ± 0.0	100.0 ± 0.0	95.4 ± 0.6
internal steric clash	96.8 ± 0.2	96.7 ± 0.6	94.4 ± 1.1	92.2 ± 0.4
aromatic ring flatness	100.0 ± 0.0	100.0 ± 0.0	100.0 ± 0.0	100.0 ± 0.0
non-aromatic ring non-flatness	99.9 ± 0.0	99.7 ± 0.1	99.8 ± 0.2	97.3 ± 0.3
double bond flatness	99.7 ± 0.1	99.2 ± 0.2	99.4 ± 0.2	99.9 ± 0.1
internal energy	100.0 ± 0.0	99.9 ± 0.1	99.9 ± 0.1	94.4 ± 0.3
all	95.9 ± 0.2	88.5 ± 1.3	86.6 ± 0.7	82.7 ± 0.8

S2 Example Molecules

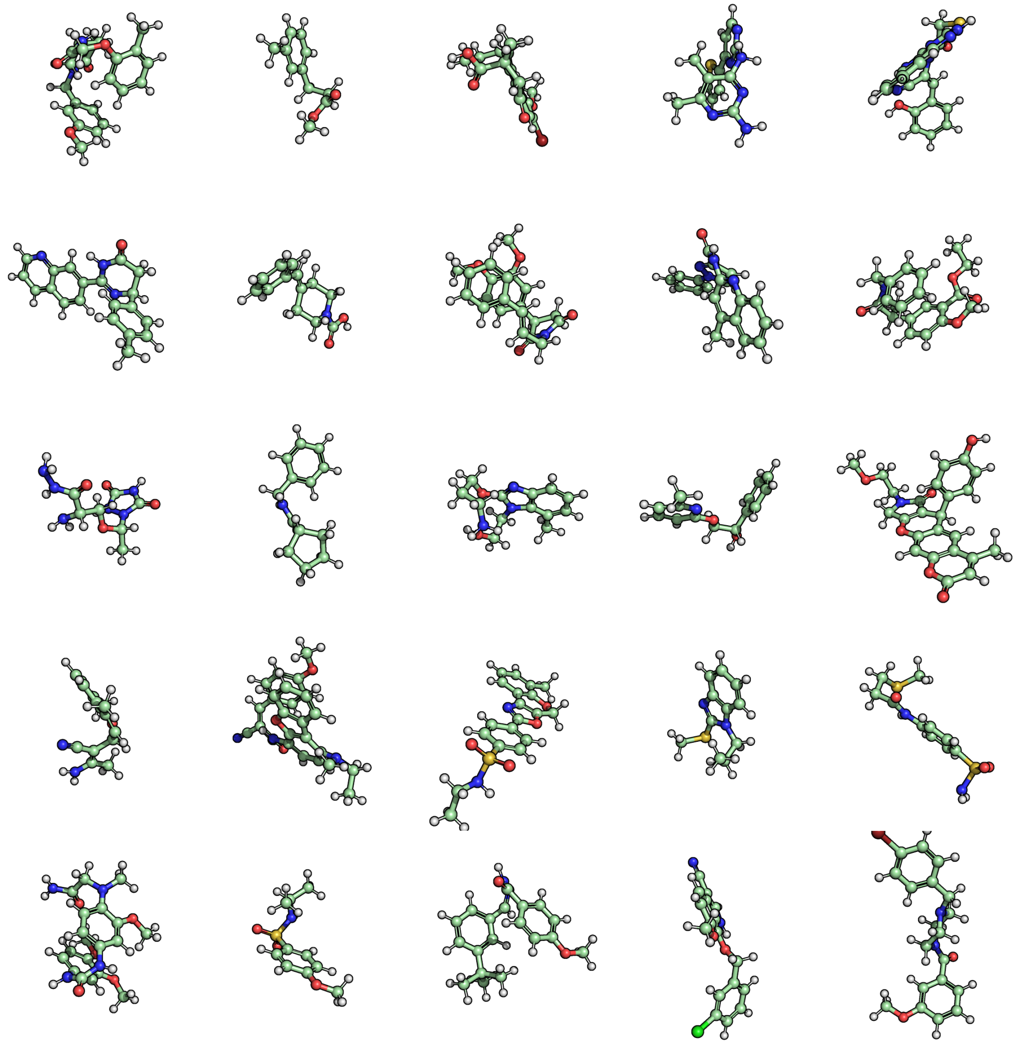

Figure S1: Example molecules sampled from FlowMol3

S3 The Coupling Distribution

Since we chose the conditioning variable for our conditional probability paths to be pairs of initial and final molecules (g_0, g_1), we must also define a method

of obtaining such paired samples of initial and final molecules. In other words, we must define the distribution from which our conditioning variable is obtained $p(g_0, g_1)$. This is generally referred to as a coupling distribution[2–4].

The simplest choice of the coupling distribution would be an independent coupling:

$$p(g_0, g_1) = p(g_0)p(g_1) \tag{1}$$

In FlowMol, the coupling distribution factorizes over modalities.

$$p(g_0, g_1) = p(X_0, X_1)p(A_0, A_1)p(C_0, C_1)p(E_0, E_1) \tag{2}$$

All discrete modalities are given the independent coupling:

$$p(A_0, A_1) = p(A_0)p(A_1) \tag{3}$$

That is, the the target value (A_1) is obtained from the dataset, and the corresponding prior value (A_0) is always a sequence of mask tokens, independent of the target value.

However, in continuous flow matching, the independent coupling can introduce pathologies that impair model performance such as having a large transport cost (distances between prior and target samples), and intersecting conditional paths. Several works identify this pathology and propose various forms of coupling distributions that involve drawing independent samples and then aligning them in some fashion[3,5,6].

For continuous modalities, we implement a technique similar to the Equivariant Optimal Transport Coupling proposed by Klein *et al.*[6]. We first obtain the $t = 0$ atom coordinates as independent samples from a standard Gaussian $X_0 \sim \prod_{i=1}^{N} \mathcal{N}(x_0^i|0, \mathbb{I}_3)$. We then align the prior coordinates X_0 to the ground-truth coordinates X_1 via the Kabsch algorithm. Next, we permute the order of atoms in X_0. This can be thought of as permuting the intial positions of each atom. We specifically choose the permutation that minimizes the sum of distances between initial and final positions across all atoms (this is known as solving the assignment problem).

S4 Training Details and Hyperparameters

FlowMol3 is trained with 6 Molecule Update Blocks. Atoms contain 256 hidden scalar features and 32 hidden vector features. Edges contain 128

hidden features. All models are trained for 20 epochs. GEOM models are trained on 4xL40 GPUs with an adaptive batch-size that typically yields 15–20 graphs per batch per GPU. Training takes approximately 4–5 days.

As described in Section 2.4 of the main paper, the overall loss for training FlowMol3 is a weighted sum of per-modality flow matching losses. The loss weights $(\lambda_X, \lambda_A, \lambda_C, \lambda_E)$ are set to (3, 0.4, 1, 2).

S4.1 Optimizer and Training Configuration

Table S2 summarizes the optimizer settings, batch configuration, and learning rate schedule used for training FlowMol3.

Table S2: Training Configuration for FlowMol3

**Parameter**	**Value**
Optimizer	
Optimizer	AdamW
Base learning rate	1×10^{-4}
Weight decay	1×10^{-12}
Learning Rate Schedule	
Schedule type	Constant (no decay)
Warmup steps	None
Batch Configuration	
Batch sizing method	Adaptive (edge-based)†
Max edges per batch	50,000
Typical batch size (per GPU)	∼17 graphs
Number of GPUs	4× NVIDIA L40
Gradient accumulation steps	2
Effective batch size	∼136 graphs
Regularization	
EMA decay	0 (disabled)
Gradient clipping	None
Training Duration	
Epochs	20
Distributed strategy	DDP

†Batch size is determined adaptively using a custom PyTorch sampler: graphs are added to each batch until the cumulative number of edges exceeds the threshold. Since molecular graphs are modeled as fully connected (number of edges $\approx N^2$ for a molecule with N atoms), this approach ensures consistent GPU memory usage across batches regardless of molecule size variation. The expected number of edges per molecule in the GEOM dataset is approximately 3,000, yielding roughly 50,000/3,000 $\approx$ 17 graphs per batch per GPU.

S4.2 Model Architecture Hyperparameters

Table S3 provides the complete architecture hyperparameters for the FlowMol3 neural network.

Table S3: FlowMol3 Architecture Hyperparameters

**Parameter**	**Value**
Model Structure	
Molecule Update Blocks	6
Convolutions per update	1
Recycles	1
Feature Dimensions	
Hidden scalar features (atoms)	256
Hidden vector features (atoms)	32
Hidden edge features	128
Atom/charge/edge token dim	64
Time embedding dim	64
Attention heads	32
GVP Configuration	
Expansion GVPs	3
Message GVPs	3
Update GVPs	3
Cross-product features	4
Distance Encoding	
RBF dimension	32
RBF max distance	10 Å
Self-Conditioning	
Self-conditioning	Enabled
Self-conditioning proportion	0.50
High confidence threshold	0.9

S4.3 Flow Matching Hyperparameters

Table S4 lists the hyperparameters specific to the flow matching training procedure.

Table S4: Flow Matching Training Hyperparameters

**Parameter**	**Value**
Loss Weights	
Coordinate loss weight (λ_X)	3.0
Atom type loss weight (λ_A)	0.4
Charge loss weight (λ_C)	1.0
Bond loss weight (λ_E)	2.0
Interpolant Schedule	
Coordinate schedule	Linear
Atom type schedule	Linear
Charge schedule	Linear
Bond schedule	Linear
Prior Configuration	
Coordinate prior	Centered normal ($\sigma = 1.0$)
Discrete priors (A, C, E)	CTMC (mask token)
Coordinate alignment	Kabsch + optimal assignment
Data Augmentation	
Geometry distortion prob. (p_{distort})	0.70
Distortion time threshold (t_{distort})	0.25
Fake atom probability (p_{fake})	0.30
Fake atom anchor std (σ_{fake})	1.0

All model hyperparameters are visible in the config files provided in our GitHub repository.

S5 Additional Techniques for DFM Sampling

Here we discuss three techniques we apply to sampling discrete modalities in FlowMol3: remasking, low-temperature sampling, and purity sampling.

S5.1 Remasking via Stochasticity

Recall our marginal probability velocity ((12) from the main paper, reproduced here)

$$u^i(j, A_t) = \frac{1+\eta t}{1-t} p^{\theta}_{1|t}(a^i_1 = j|A_t)\delta_M(a^i_t) + \eta(1 - \delta_M(a^i_t))\delta_M(j) \quad (4)$$

Includes the hyperparameter $\eta \geq 0$ which can be chosen at inference time. η can be called the "stochasticity parameter". If we set $\eta = 0$ then once a sequence element (i.e., an atom type or a bond order) is unmasked, that value is fixed for the remainder of the trajectory. If we choose $\eta > 0$, sequence elements may be remasked and unmasked again repeatedly throughout a trajectory; enabling the denoiser to correct or change past decisions. We find that enabling remasking significantly enhances sample quality. All FlowMol results obtained in this paper are done so using $\eta = 30$.

S5.2 Low-Temperature Sampling

Recall that for discrete modalities our neural networks directly approximate the distribution of states at $t = 1$ for each sequence element. In the context of atom types, for example, this quantity is denoted $p^{\theta}_{1|t}(a^i_1|A_t)$.

We re-normalize logits obtained from the model using a fixed temperature τ:

$$p^{\theta}_{1|t}(a^i_1|A_t) = \text{softmax}\left(\tau^{-1} \log p^{\theta}_{1|t}(a^i_1|A_t)\right) \quad (5)$$

We find that low-temperature sampling, which biases the sampled discrete states towards the most confident ones, to be critical for model performance. In practice we use $\tau = 0.05$.

S5.3 Purity Sampling

Under the base DFM formulation, at each integration step, the probability of unmasking each currently-masked sequence element is $\Delta t \frac{1+\eta t}{1-t}$. Every masked token has an equal probability of getting unmasked.

Rather than doing this, we implement purity sampling as described in Campbell *et al.*[7]. We select which currently-masked elements to unmask using a proxy for model confidence. The proxy used for model confidence is the "purity": the maximum category probability output by the model.

During inference, at each integration step, we first sample the number of elements to unmask from a binomial distribution with n equal to the

number of mask tokens in the sequence and $p = \Delta t \frac{1+\eta t}{1-t}$. Then after we obtain $k \sim \text{Binomial}(n, p)$, we select the top-$k$ sequence elements with the highest "purity"; these are the tokens that will be unmasked.

S6 GVP with Cross Product

A geometric vector perception (GVP) can be thought of as a single-layer neural network that applies linear and point-wise non-linear transformation to its inputs. The difference between a GVP and a conventional feed-forward neural network is that GVPs operate on two distinct data types: scalars and vectors. GVPs also allow these data types to exchange information while preserving equivariance of the output vectors. The original GVP only applied linear transformations to the vector features and as a result produces output vectors that are E(3)-equivariant.

We introduce a modification to the GVP as its presented in Jing *et al.*[8]; specifically we perform a cross product operation on the input vectors. The motivation for this is that the cross product is *not* equivariant to reflections. We refer the reader to Appendix F of Schneuing *et al.*[9] for a detailed discussion of the equivariance of cross products. As a result, the version of GVP we present here is SE(3) equivariant. The benefit of being SE(3) equivariant rather than E(3) equivariant is that the generative model becomes sensitive to chiral centers in molecules, since reflecting the molecule will produce different model outputs. The operations for our cross product enhanced GVP are described in Algorithm 1.

S7 Examples of Out-of-Distribution Ring Systems

To provide intuition about the purpose or significance of the OOD ring system metric, we provide examples of OOD ring systems produced by evaluated models in Figure S2.

Algorithm 1 Geometric Vector Perceptron with Cross Product

Input: Scalar and vector features: $(s, v) \in \mathbb{R}^f \times \mathbb{R}^{\nu \times 3}$
Output: Scalar and vector features: $(s', v') \in \mathbb{R}^j \times \mathbb{R}^{\mu \times 3}$
Hyperparameter: Number of hidden vector features $n_h \in \mathbb{Z}^+$
Hyperparameter: Number of cross product features $n_{cp} \in \mathbb{Z}^+$

$v_h \leftarrow W_h v \quad \in \mathbb{R}^{n_h \times 3}$
$v_{cp} \leftarrow W_{cp} v \quad \in \mathbb{R}^{2n_{cp} \times 3}$
$v_{cp} \leftarrow v_{cp}[: n_{cp}] \times v_{cp}[n_{cp} :] \quad \in \mathbb{R}^{n_{cp} \times 3}$ // cross product
$v_{h+cp} \leftarrow \text{Concat}(v_h, v_{cp}) \quad \in \mathbb{R}^{(n_h + n_{cp}) \times 3}$
$v_\mu \leftarrow W_\mu v_{h+cp} \quad \in \mathbb{R}^{\mu \times 3}$
$s_{h+cp} \leftarrow \|v_{h+cp}\| \quad \in \mathbb{R}^{n_h + n_{cp}}$
$s_{f+h+cp} \leftarrow \text{Concat}(s, s_{h+cp})$'
$s_j \leftarrow W_j s_{f+h+cp} + b_j \quad \in \mathbb{R}^j$
$s' \leftarrow \sigma(s_j) \quad \in \mathbb{R}^j$
$v' \leftarrow \sigma_g \left(W_g[\sigma^+(s_m)] + b_g \right) \odot v_\mu$ (row-wise) $\quad \in \mathbb{R}^{\mu \times 3}$

return (s', v')

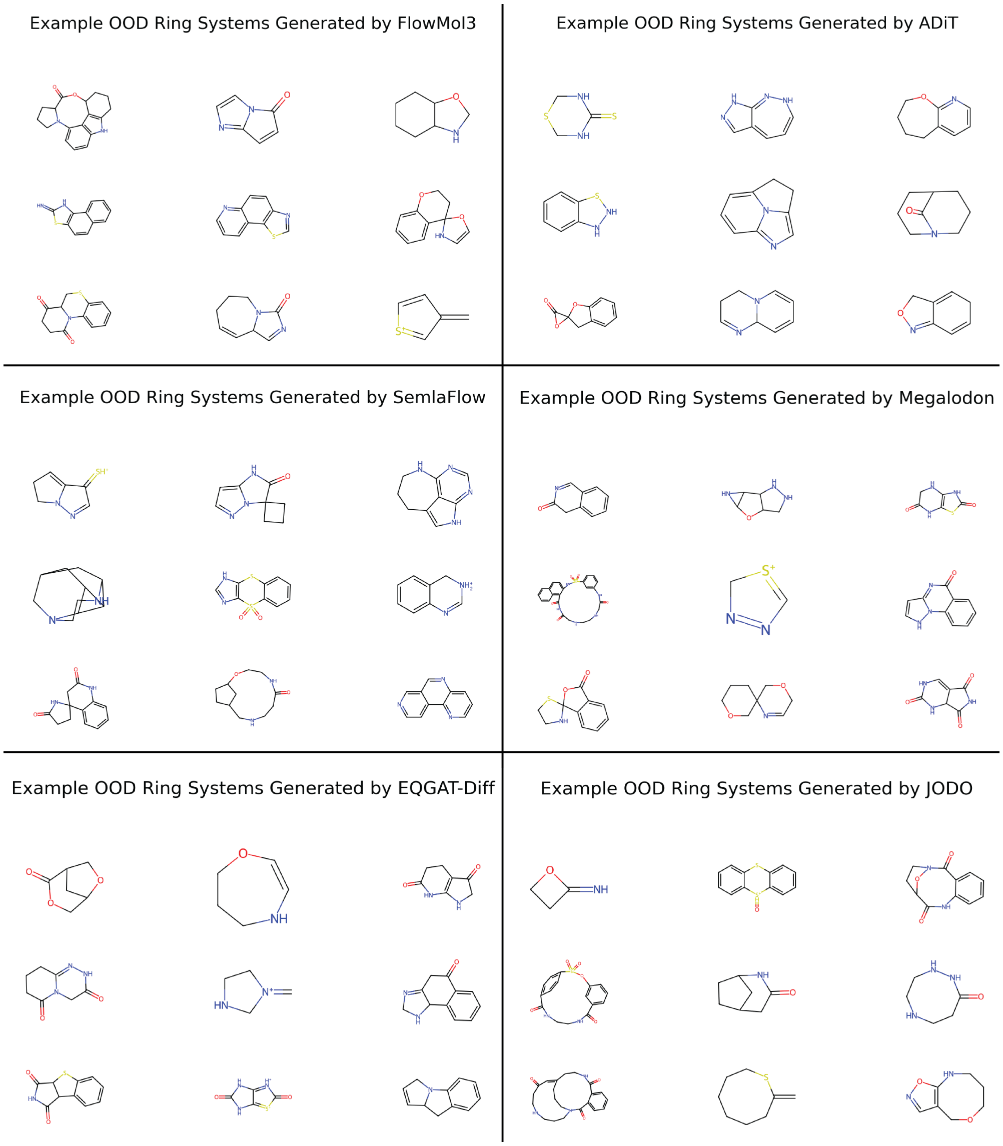

Figure S2: Examples of OOD ring systems (ring systems that do not appear in ChEMBL) that were produced by several of the models evalauted in this paper. The specific OOD ring systems were selected randomly from the set of all OOD ring systems found.

S8 Individual Functional Group Frequencies

We show the functional group frequencies for the 36 most commonly occurring functional groups evaluated in the following figures. The y-axis in all figures is the frequency of the functional group per 100 sampled molecules.

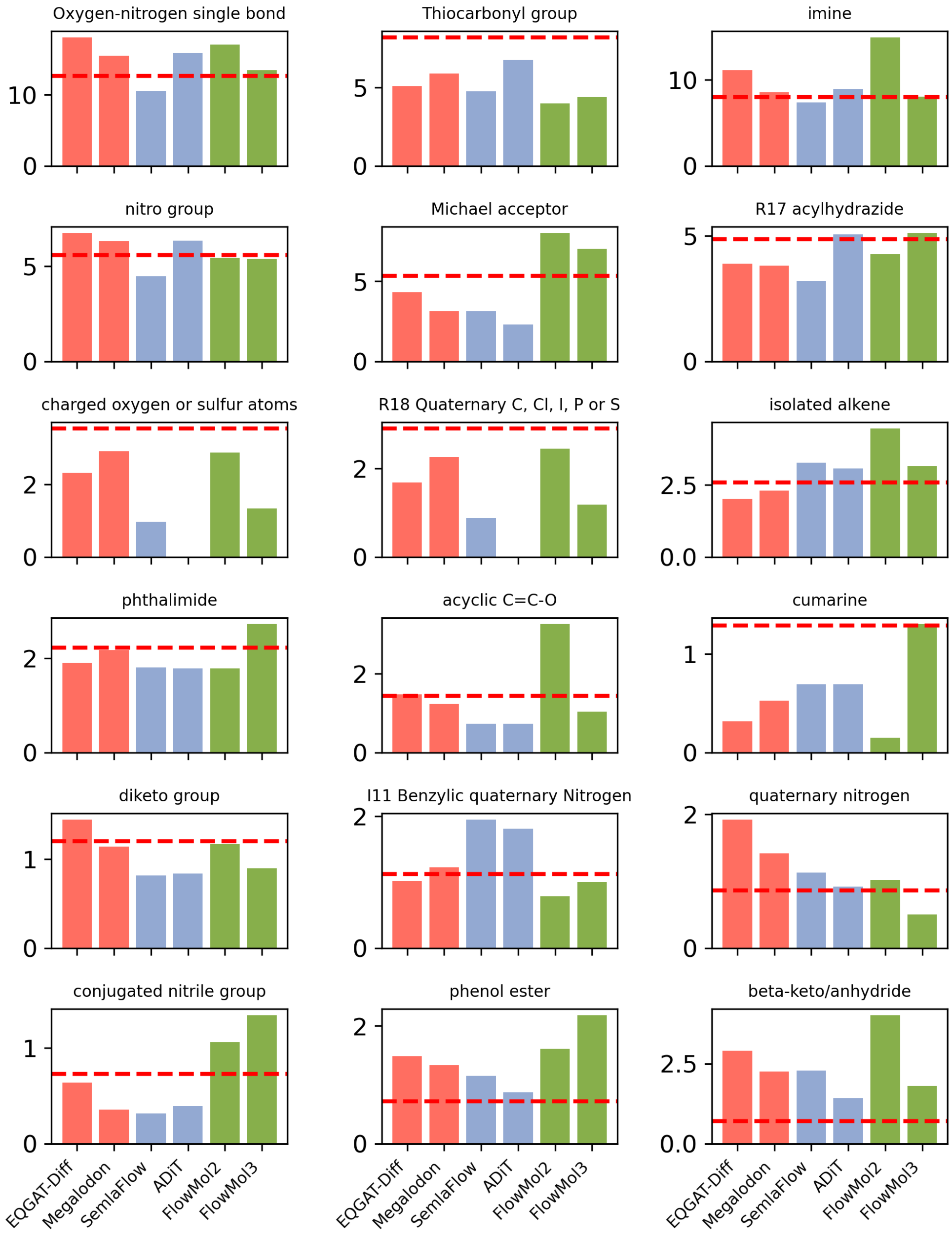
Oxygen-nitrogen single bond
Thiocarbonyl group
imine
nitro group
Michael acceptor
R17 acylhydrazide
charged oxygen or sulfur atoms
R18 Quaternary C, Cl, I, P or S
isolated alkene
phthalimide
acyclic C=C-O
cumarine
diketo group
I11 Benzylic quaternary Nitrogen
quaternary nitrogen
conjugated nitrile group
phenol ester
beta-keto/anhydride
EQGAT-Diff
Megalodon
SemlaFlow
ADiT
FlowMol2
FlowMol3

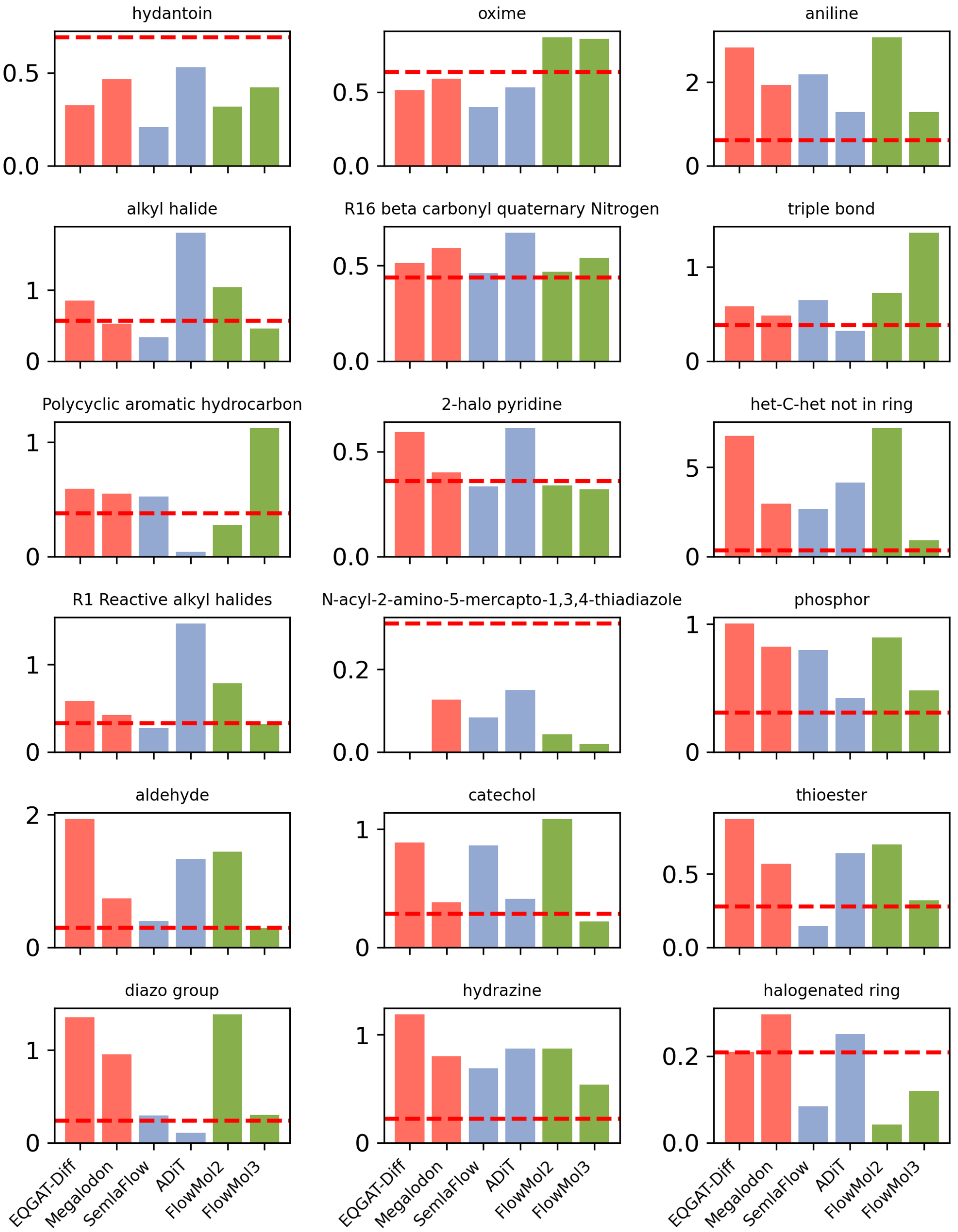

hydantoin
oxime
aniline
alkyl halide
R16 beta carbonyl quaternary Nitrogen
triple bond
Polycyclic aromatic hydrocarbon
2-halo pyridine
het-C-het not in ring
R1 Reactive alkyl halides
N-acyl-2-amino-5-mercapto-1,3,4-thiadiazole
phosphor
aldehyde
catechol
thioester
diazo group
hydrazine
halogenated ring
EQGAT-Diff
Megalodon
SemlaFlow
ADiT
FlowMol2
FlowMol3

S9 Self-Conditioning Proportion Ablation

Self-conditioning[10] is a technique where, during training, the model's own prediction from a previous forward pass is fed back as additional input. The proportion of training steps that use self-conditioning (as opposed to receiving a zero-initialized placeholder) is a hyperparameter. Following prior work, we use a self-conditioning proportion of 0.50 in our main experiments. Here we present an ablation study over this hyperparameter.

Table S5: Ablation of self-conditioning proportion with 95% confidence intervals. The first row shows reference values from the training data distribution.

Self-Cond Prop	% PB-Valid (↑)	FG Dev. (↓)	OOD Ring Rate (↓)	Med. ΔE_{relax} (↓)	Med. RMSD (↓)
Data	93.2 ± 1.0	—	0.05 ± 0.00	—	—
0.25	$\mathbf{93.9 \pm 0.2}$	0.27 ± 0.01	$\mathbf{0.09 \pm 0.00}$	$\mathbf{3.73 \pm 0.04}$	0.43 ± 0.01
0.50	91.9 ± 0.7	0.27 ± 0.03	0.10 ± 0.01	3.83 ± 0.08	0.39 ± 0.01
0.75	93.9 ± 0.6	$\mathbf{0.26 \pm 0.01}$	0.12 ± 0.00	4.23 ± 0.06	$\mathbf{0.48 \pm 0.01}$

The results in Table S5 demonstrate that FlowMol3 is relatively robust to the choice of self-conditioning proportion across the tested range. Key observations include:

- **PB-Validity:** All three proportions achieve similar validity rates (∼92–94%), with no statistically significant differences between conditions.
- **Functional Group Deviation:** Performance is consistent across all proportions (∼0.26–0.27), with the highest proportion (0.75) achieving marginally better results.
- **OOD Ring Rate:** Lower self-conditioning proportions yield fewer out-of-distribution ring systems, with 0.25 achieving the best result (0.09).
- **Relaxation Energy:** The median relaxation energy increases with self-conditioning proportion, from 3.73 kcal/mol at 0.25 to 4.23 kcal/mol at 0.75—a difference of 0.50 kcal/mol. This suggests that lower proportions may produce slightly better initial geometries.

- **Ring Rate:** The 0.50 proportion achieves the highest ring rate (2.16), closest to the training data distribution (2.46), indicating better capture of ring system prevalence.

Overall, these results support the use of the conventional 0.50 proportion as a reasonable default that balances performance across multiple metrics. The method demonstrates robustness to this hyperparameter, with all tested values producing high-quality molecules.

S10 Geometry Distortion Hyperparameter Ablation

FlowMol3 employs a late-stage geometry distortion technique where noise is applied to a random subset of atom coordinates during training. This is controlled by two hyperparameters: p_{distort} (the probability that any given atom receives distortion) and t_{distort} (the timestep threshold above which distortion is applied). Here we present ablation studies over these hyperparameters.

Table S6: Ablation of geometry distortion hyperparameters with 95% confidence intervals. The first row shows reference values from the training data distribution.

p_{distort}	t_{distort}	% PB-Valid (↑)	FG Dev. (↓)	OOD Ring Rate (↓)	Med. ΔE_{relax} (↓)	Med. RMSD (↓)	Ring Rate (↑)
—	—	93.2 ± 1.0	—	0.05 ± 0.00	—	—	2.46 ± 0.00
1.00	0.00	$\mathbf{99.1 \pm 0.1}$	0.67 ± 0.02	0.07 ± 0.00	3.46 ± 0.07	$\mathbf{0.04 \pm 0.00}$	1.77 ± 0.00
0.70	0.25	95.9 ± 0.2	0.37 ± 0.01	$\mathbf{0.05 \pm 0.00}$	4.50 ± 0.07	0.28 ± 0.01	1.93 ± 0.00
0.70	0.50	95.0 ± 0.4	0.34 ± 0.02	0.12 ± 0.00	4.45 ± 0.04	0.34 ± 0.01	1.65 ± 0.00
0.20	0.25	95.9 ± 0.4	0.31 ± 0.02	0.07 ± 0.00	$\mathbf{2.54 \pm 0.03}$	0.37 ± 0.01	1.74 ± 0.00
0.20	0.50	91.9 ± 0.7	$\mathbf{0.27 \pm 0.03}$	0.10 ± 0.01	3.83 ± 0.08	0.39 ± 0.01	$\mathbf{2.16 \pm 0.00}$
0.20	0.75	93.4 ± 0.6	0.33 ± 0.01	0.16 ± 0.00	4.03 ± 0.05	0.45 ± 0.00	1.91 ± 0.00
0.10	0.50	93.2 ± 0.2	0.31 ± 0.02	0.15 ± 0.01	3.57 ± 0.06	0.46 ± 0.01	1.86 ± 0.00

The distortion hyperparameters interact in complex ways, but several patterns emerge from Table S6:

- **PB-Validity:** The highest validity (99.1%) is achieved with extreme distortion ($p_{\text{distort}} = 1.0$, $t_{\text{distort}} = 0.0$), but this comes at the cost of poor functional group fidelity (FG Dev. = 0.67). At moderate p_{distort} values (0.20–0.70), validity remains high (92–96%) while FG deviation improves substantially.

- **Functional Group Deviation:** Lower p_{distort} values consistently produce better FG deviation, with the best result (0.27) at $p_{\text{distort}} = 0.20$, $t_{\text{distort}} = 0.50$. This suggests that less aggressive distortion allows the model to better learn functional group patterns.

- **OOD Ring Rate:** Lower t_{distort} values (0.00–0.25) produce fewer out-of-distribution ring systems across all p_{distort} levels. Higher t_{distort} (0.50–0.75) consistently increases OOD ring rate.

- **Relaxation Energy:** The best geometry quality (lowest $\Delta E_{\text{relax}} =$ 2.54 kcal/mol) is achieved at $p_{\text{distort}} = 0.20$, $t_{\text{distort}} = 0.25$. Higher p_{distort} values tend to produce worse geometries.

Overall, the distortion hyperparameters present a clear trade-off: aggressive distortion ($p_{\text{distort}} = 1.0$, $t_{\text{distort}} = 0.0$) maximizes PB-validity (99.1%) but dramatically worsens functional group fidelity and may reduce sample diversity. More conservative settings ($p_{\text{distort}} = 0.20$) yield better FG deviation but lower validity. We chose $p_{\text{distort}} = 0.70$, $t_{\text{distort}} = 0.25$ for the final model as it achieves high validity (95.9%), the best OOD ring rate (0.05, matching the training data), and good geometric quality (Med. RMSD = 0.28 Å), while accepting a modest increase in FG deviation (0.37) as an acceptable trade-off.

To provide further insight into how distortion hyperparameters affect functional group generation, Figure S3 shows the occurrence rates for 16 representative functional groups across four distortion settings. The red dashed line indicates the training data rate. These results demonstrate that more aggressive distortion tends to systematically under-generate most functional groups, while the chosen $(0.7, 0.25)$ setting balances validity with functional group representation.

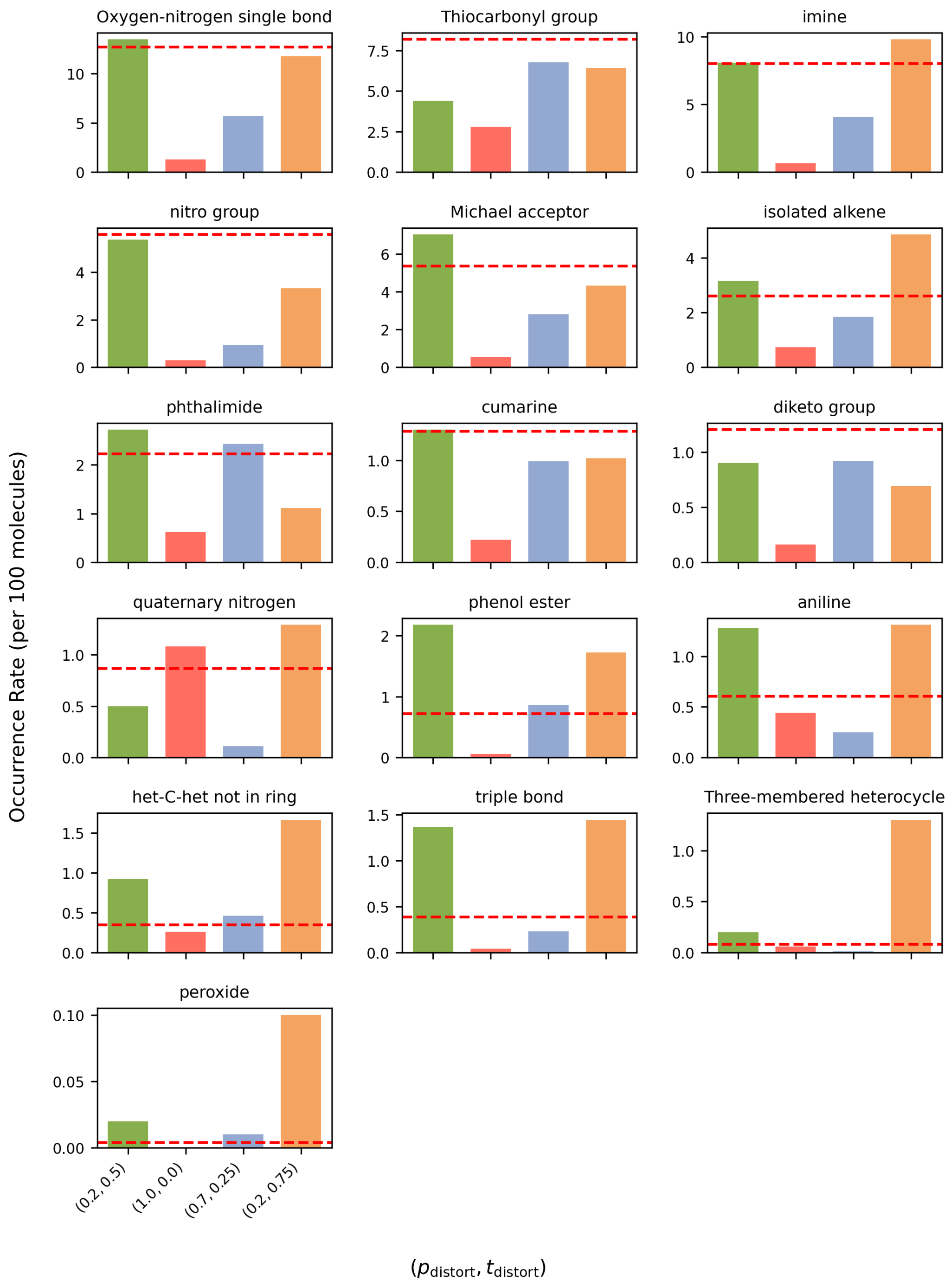

Figure S3: Functional group occurrence rates across distortion hyperparameter settings $(p_{\text{distort}}, t_{\text{distort}})$. Red dashed lines indicate training data rates. The current default $(0.7, 0.25)$ balances validity with functional group representation, while extreme distortion $(1.0, 0.0)$ tends to under-generate most functional groups.

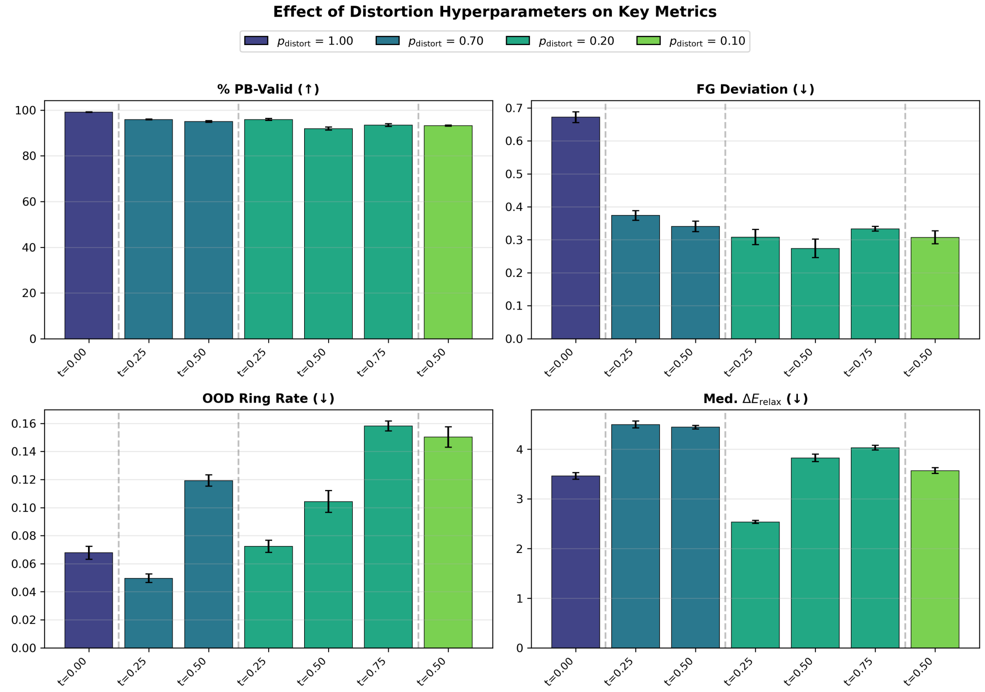

Figure S4: **Distortion Hyperparameter Sensitivity Analysis.** Effect of varying p_{distort} (color) and t_{distort} (x-axis) on four key metrics. Vertical dashed lines separate groups with different p_{distort} values.

S11 Loss Weight Sensitivity Analysis

The per-modality loss weights $(\lambda_X, \lambda_A, \lambda_C, \lambda_E)$ control the relative importance of each modality during training. We performed a 3-point sensitivity sweep over each loss weight, starting from the baseline values (3, 0.4, 1.0, 2.0) and doubling/halving each parameter individually.

Table S7: Ablation of modality loss weights with 95% confidence intervals.

λ_x	λ_a	λ_c	λ_e	% PB-Valid (↑)	FG Dev. (↓)	OOD Ring Rate (↓)	Med. ΔE_{relax} (↓)	Ring Rate (↑)
1.50	0.40	1.00	2.00	$\mathbf{95.5 \pm 0.6}$	0.28 ± 0.01	0.10 ± 0.01	4.33 ± 0.05	1.72 ± 0.00
3.00	0.40	2.00	2.00	94.9 ± 0.3	0.34 ± 0.02	$\mathbf{0.07 \pm 0.01}$	3.43 ± 0.05	1.77 ± 0.00
3.00	0.80	1.00	2.00	94.8 ± 0.3	0.28 ± 0.02	0.11 ± 0.00	3.71 ± 0.05	1.83 ± 0.00
3.00	0.20	1.00	2.00	94.5 ± 0.2	0.39 ± 0.02	0.23 ± 0.00	4.25 ± 0.06	1.54 ± 0.00
6.00	0.40	1.00	2.00	93.5 ± 0.3	0.31 ± 0.02	0.09 ± 0.01	$\mathbf{2.81 \pm 0.04}$	1.83 ± 0.00
3.00	0.40	1.00	4.00	93.4 ± 0.3	0.33 ± 0.01	0.15 ± 0.00	3.56 ± 0.07	1.89 ± 0.00
3.00	0.40	1.00	1.00	92.1 ± 0.7	0.32 ± 0.02	0.08 ± 0.01	3.74 ± 0.05	1.70 ± 0.00
3.00	0.40	1.00	2.00	91.9 ± 0.7	$\mathbf{0.27 \pm 0.03}$	0.10 ± 0.01	3.83 ± 0.08	$\mathbf{2.16 \pm 0.00}$
3.00	0.40	0.50	2.00	91.6 ± 0.2	0.37 ± 0.02	0.11 ± 0.00	3.40 ± 0.04	1.83 ± 0.00

Key observations from Table S7:

- **Coordinate loss weight (λ_x):** Increasing λ_x strongly reduces median relaxation energy, indicating improved geometry quality, but has minimal effect on validity or OOD ring rate.
- **Atom type loss weight (λ_a):** Higher λ_a values correlate with lower relaxation energy and reduced OOD ring rate, suggesting that emphasizing atom type prediction improves both geometric and chemical validity.
- **Charge loss weight (λ_c):** This parameter shows the strongest effect on PB-validity and OOD ring rate, indicating that accurate charge prediction is critical for generating chemically plausible molecules.
- **Bond loss weight (λ_e):** Increasing λ_e improves PB-validity but increases OOD ring rate, suggesting a trade-off between bond accuracy and novel ring system generation.

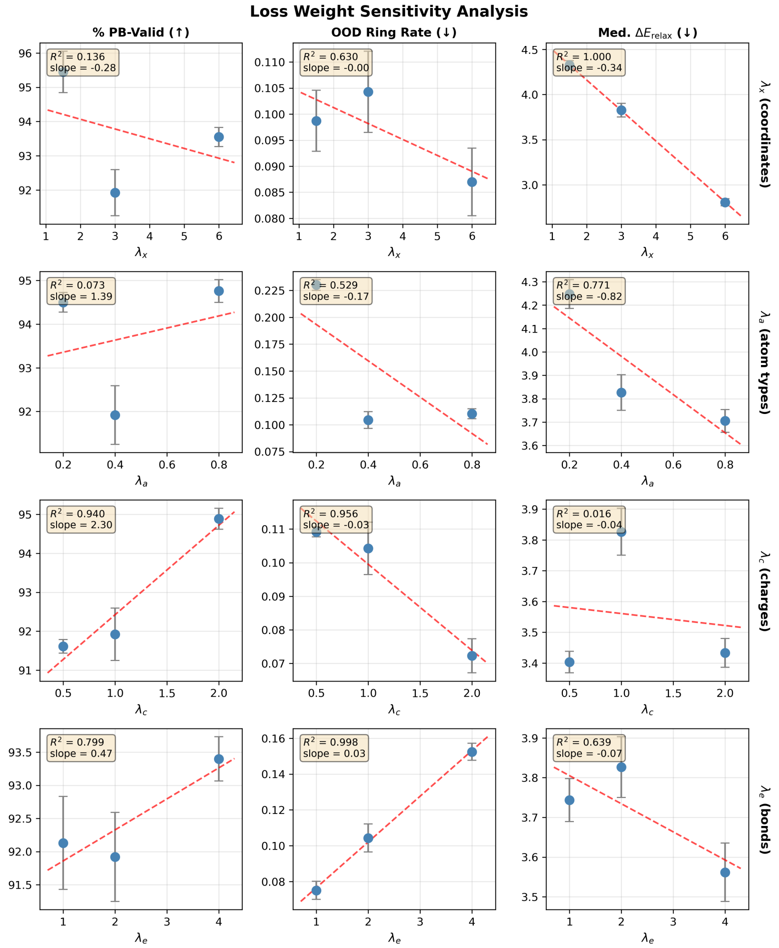

Figure S5: **3-point Sensitivity Sweep on Modality Loss Weights.** Effect of changing each modality loss weight on three metrics: PoseBusters validity, OOD ring rate, and the median energy of relaxation.

S12 Fake Atom Hyperparameter Ablation

FlowMol3 uses "fake atoms" during training—additional atoms that are added to molecules and must be identified and removed by the model. This technique is controlled by two hyperparameters: p_{fake} (the probability of adding a fake atom per real atom) and σ_{fake} (the standard deviation of the anchor position for fake atoms). Here we present ablation studies over these

hyperparameters.

Table S8: Ablation of fake atom hyperparameters with 95% confidence intervals. The first row shows reference values from the training data distribution.

p_{fake}	σ_{fake}	% PB-Valid (↑)	FG Dev. (↓)	OOD Ring Rate (↓)	Med. ΔE_{relax} (↓)	Med. RMSD (↓)	Ring Rate (↑)
—	—	93.2 ± 1.0	—	0.05 ± 0.00	—	—	2.46 ± 0.00
0.10	1.00	90.5 ± 0.4	0.32 ± 0.01	0.14 ± 0.01	4.15 ± 0.07	$\mathbf{0.50 \pm 0.01}$	1.97 ± 0.00
0.30	0.25	94.2 ± 0.1	$\mathbf{0.23 \pm 0.01}$	0.13 ± 0.01	$\mathbf{3.01 \pm 0.04}$	0.38 ± 0.01	1.86 ± 0.00
0.30	1.00	91.9 ± 0.7	0.27 ± 0.03	$\mathbf{0.10 \pm 0.01}$	3.83 ± 0.08	0.39 ± 0.01	$\mathbf{2.16 \pm 0.00}$
0.30	2.50	$\mathbf{94.3 \pm 0.7}$	0.26 ± 0.01	0.14 ± 0.01	3.93 ± 0.03	0.41 ± 0.01	1.71 ± 0.00
0.70	1.00	93.2 ± 0.8	0.34 ± 0.01	0.11 ± 0.00	3.90 ± 0.14	0.47 ± 0.01	1.55 ± 0.00

Key observations from Table S8:

- **Fake atom probability (p_{fake}):** Increasing the fake atom probability shows a positive correlation with PB-validity, suggesting that higher fake atom rates improve molecular validity. The OOD ring rate decreases slightly with higher p_{fake}.
- **Anchor position standard deviation (σ_{fake}):** This parameter shows the strongest effect on relaxation energy, with lower values producing better geometries (3.01 kcal/mol at $\sigma = 0.25$ vs. 3.93 kcal/mol at $\sigma = 2.5$). PB-validity and OOD ring rate show minimal sensitivity to this parameter.

Overall, the fake atom probability has the most pronounced effect on molecular validity, while the anchor standard deviation primarily influences geometry quality. The baseline values ($p_{\text{fake}} = 0.3$, $\sigma_{\text{fake}} = 1.0$) represent a reasonable trade-off across metrics.

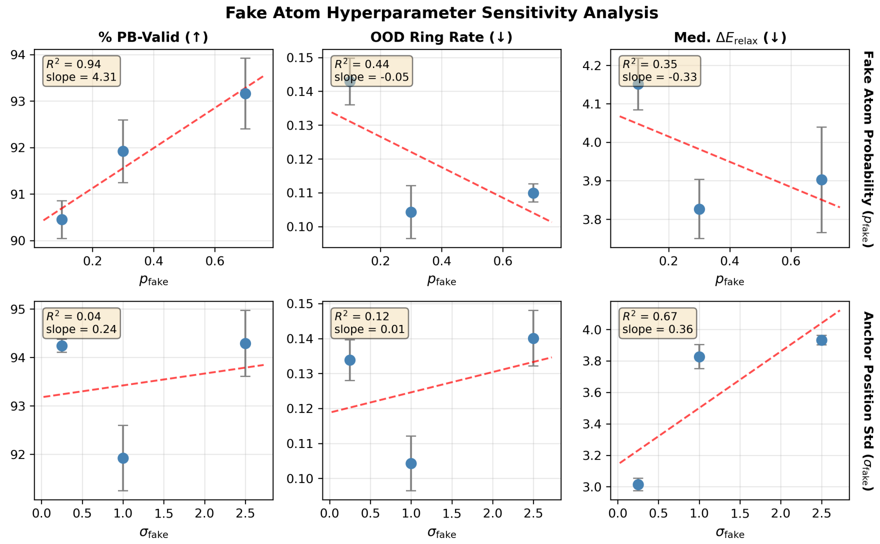

Figure S6: **Fake Atom Hyperparameter Sensitivity Analysis.** Effect of varying the fake atom probability p_{fake} (top row, with $\sigma_{\text{fake}} = 1.0$ fixed) and anchor position standard deviation σ_{fake} (bottom row, with $p_{\text{fake}} = 0.3$ fixed) on three key metrics.

S13 Ring System Statistics

Table S9 presents ring system statistics comparing FlowMol3 samples with the training data distribution. We report the mean count per molecule of ring systems categorized by their fusion level: monocyclic (single rings), bicyclic (fused-2), and tricyclic (fused-3) ring systems. We also include results for FlowMol3 variants trained with different geometry distortion hyperparameters to illustrate how these settings affect ring system generation.

Table S9: Ring System Statistics: Mean count per molecule of ring systems by ring fusion level. Single = monocyclic ring systems, Fused-2/3 = bicyclic/tricyclic fused ring systems.

Model	Total (↑)	Single (↑)	Fused-2 (↑)	Fused-3 (↑)
Training Data	2.46	2.01	0.37	0.06
FlowMol3	**2.16**	**1.68**	**0.39**	**0.07**
FlowMol3 (p=0.7, t=0.25)	1.93	1.56	0.32	0.05
FlowMol3 (p=1.0, t=0.0)	1.77	1.47	0.22	0.04

FlowMol3 generates molecules with ring system distributions that closely match the training data. The baseline FlowMol3 model produces 2.16 ring systems per molecule on average, compared to 2.46 in the training data—a modest 12% reduction. The distribution across fusion levels is well-preserved: FlowMol3 generates slightly fewer monocyclic rings (1.68 vs. 2.01) while matching or slightly exceeding the training data rates for fused ring systems (0.39 vs. 0.37 for bicyclic, 0.07 vs. 0.06 for tricyclic).

Increasing the geometry distortion hyperparameters (p_{distort} and reducing t_{distort}) leads to progressively lower ring system rates across all fusion levels. This is expected, as stronger distortion during training encourages the model to focus on learning robust coordinate predictions rather than memorizing specific ring geometries.

S14 Speed–Quality Trade-off

Figure S7 shows how molecule quality metrics scale with the number of integration steps used during sampling. Inset panels provide a zoomed view of the asymptotic behavior at higher step counts.

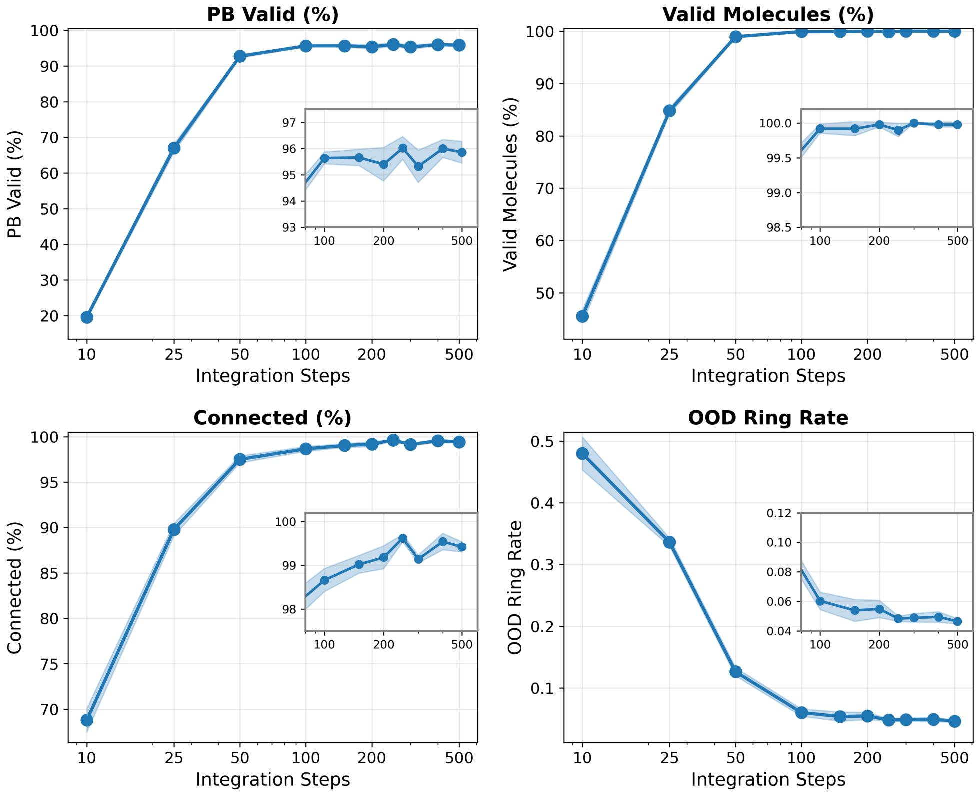

Figure S7: **Speed–Quality Trade-off.** Four metrics of molecule quality as a function of integration steps. Main panels show the full range; inset panels zoom into the high-step region (100–500) to reveal asymptotic behavior. Shaded regions indicate 95% confidence intervals.

Key observations:

- **Rapid initial improvement:** All metrics show dramatic improvement between 10 and 50 integration steps. PB-validity increases from $\sim$20% to $\sim$93%, valid molecule fraction rises from $\sim$46% to $\sim$99%, and connectivity improves from $\sim$69% to $\sim$98%.
- **Diminishing returns beyond 100 steps:** As shown in the inset panels, metrics largely plateau after 100 integration steps. PB-validity stabilizes around 95–96%, with fluctuations of $\pm$1% that fall within confidence intervals. Valid molecule and connectivity fractions reach >99% and remain stable.

- **OOD ring rate:** This metric decreases from $\sim$0.48 at 10 steps to $\sim$0.07 at 100+ steps, with minimal further improvement. The inset shows stable performance between 0.05–0.07 across the high-step range.
- **Practical recommendation:** Based on these results, 100–200 integration steps provide near-optimal molecule quality. Using more steps yields negligible improvements while increasing inference time proportionally. For applications requiring faster generation, 50 steps achieves >90% PB-validity with a 2–4× speedup.

For results in the paper we use 250 integration steps, so the quality is close to the maximum that could be obtained from FlowMol3 with our sampling method. These results demonstrate that speed gains could be achieved if the user is willing to sacrifice a small amount of quality.

S15 On the Intractability of Quantifying Distribution Drift

S15.1 Introduction and Motivation

In flow matching generative models, a neural network v_θ is trained to approximate a target vector field $u_t(x)$ that generates a probability path p_t from a source distribution p_0 to a target distribution p_1. During inference, samples are generated by integrating the learned vector field:

$$\frac{dx}{dt} = v_\theta(t, x), \quad x(0) \sim p_0. \tag{6}$$

Because $v_\theta \neq u_t$ in general, the distribution of samples generated by the learned model, which we denote p_t^θ, will differ from the theoretical marginal distribution p_t. We refer to this discrepancy as *distribution drift*. In this appendix, we provide a rigorous argument for why such quantification is fundamentally intractable within the conditional flow matching (CFM) framework.

S15.2 Background: The Flow Matching Framework

S15.2.1 Probability Paths and the Continuity Equation

Let $u_t : \mathbb{R}^d \to \mathbb{R}^d$ be a time-dependent vector field for $t \in [0, 1]$. This vector field defines an ordinary differential equation

$$\frac{dx}{dt} = u_t(x), \tag{7}$$

whose flow map $\phi_t : \mathbb{R}^d \to \mathbb{R}^d$ satisfies $\phi_0(x) = x$. Given an initial density p_0, the flow induces a time-varying density $p_t = [\phi_t]_{\#} p_0$ (the pushforward of p_0 under ϕ_t). The relationship between p_t and u_t is governed by the *continuity equation*:

$$\frac{\partial p_t}{\partial t} + \nabla \cdot (p_t u_t) = 0. \tag{8}$$

S15.2.2 Marginal Vector Fields as Intractable Expectations

In conditional flow matching, the marginal probability path is constructed as a mixture of simpler conditional paths:

$$p_t(x) = \int p_t(x \mid z)\, q(z)\, dz, \tag{9}$$

where z is a conditioning variable (typically a pair of endpoints (x_0, x_1)), $q(z)$ is a distribution over conditions, and $p_t(x \mid z)$ is a tractable conditional probability path. The corresponding marginal vector field that generates p_t is given by:

$$u_t(x) = \mathbb{E}_{q(z)} \left[\frac{u_t(x \mid z)\, p_t(x \mid z)}{p_t(x)} \right] = \frac{\int u_t(x \mid z)\, p_t(x \mid z)\, q(z)\, dz}{p_t(x)}. \tag{10}$$

Crucially, both the marginal density $p_t(x)$ in the denominator and the expectation in the numerator require integration over the entire support of $q(z)$—typically the joint distribution over all source-target pairs. These integrals are intractable for any non-trivial data distribution. This intractability is precisely what motivates the CFM framework: we train by regressing against the *conditional* vector field $u_t(x \mid z)$, which is tractable, rather than the marginal $u_t(x)$.

S15.3 Candidate Definitions of Drift

We now consider several natural mathematical definitions of "drift" and demonstrate that each is intractable to compute.

S15.3.1 Velocity Field Discrepancy

The most direct notion of approximation error is the discrepancy between the learned and true vector fields. One could define an instantaneous velocity error:

$$\mathcal{E}_v(t) := \mathbb{E}_{x\sim p_t} \|v_\theta(t,x) - u_t(x)\|^2 = \int p_t(x) \|v_\theta(t,x) - u_t(x)\|^2 \, dx. \tag{11}$$

Intractability. Computing $\mathcal{E}_v(t)$ requires:

1. Evaluating the marginal density $p_t(x)$, which by Eq. (9) requires integrating over all conditioning variables z.
2. Evaluating the marginal vector field $u_t(x)$, which by Eq. (10) requires the same intractable marginalization.

This is precisely the computation that CFM was designed to avoid. The training objective $\mathcal{L}_{\text{CFM}}(\theta) = \mathbb{E}_{t,q(z),p_t(x|z)}\|v_\theta(t,x) - u_t(x|z)\|^2$ provides unbiased gradients for minimizing $\mathcal{E}_v(t)$ without ever computing $u_t(x)$ explicitly, but this does not give us access to the value of $\mathcal{E}_v(t)$ itself.

An integrated or trajectory-averaged velocity error could be defined as:

$$\mathcal{E}_v^{\text{traj}} := \mathbb{E}_{x_0\sim p_0} \int_0^1 \left\|v_\theta(t, x^\theta(t)) - u_t(x^\theta(t))\right\|^2 dt, \tag{12}$$

where $x^\theta(t)$ is the trajectory under the learned flow. This remains intractable since $u_t(\cdot)$ cannot be evaluated pointwise.

S15.3.2 Distributional Divergence

A natural measure of drift is the divergence between the true marginal p_t and the distribution p_t^θ induced by the learned flow:

$$D_{\text{KL}}(p_t \| p_t^\theta) = \int p_t(x) \log \frac{p_t(x)}{p_t^\theta(x)} \, dx. \tag{13}$$

Intractability. This requires:

1. Access to the density $p_t(x)$, which is intractable as established above.

2. Access to the density $p_t^\theta(x)$. While we can *sample* from p_t^θ by integrating the learned ODE, computing the density requires solving an additional ODE for the log-determinant of the Jacobian of the flow (as in continuous normalizing flows), which is computationally expensive and does not help us access $p_t(x)$.

Alternative divergences such as the Wasserstein distance $W_2(p_t, p_t^\theta)$ face similar issues: computing transport costs requires access to the densities or at least the ability to sample from both distributions *with known correspondence*—but p_t cannot be sampled from directly without access to the true u_t.

S15.3.3 Accumulated Trajectory Error

One might attempt to quantify drift as the expected displacement between trajectories under the true and learned flows:

$$\mathcal{E}_{\text{traj}}(t) := \mathbb{E}_{x_0 \sim p_0} \left\| \phi_t(x_0) - \phi_t^\theta(x_0) \right\|^2, \tag{14}$$

where ϕ_t is the true flow map satisfying $\frac{d\phi_t}{dt} = u_t(\phi_t)$ and ϕ_t^θ is the learned flow map satisfying $\frac{d\phi_t^\theta}{dt} = v_\theta(t, \phi_t^\theta)$.

Intractability. Computing $\phi_t(x_0)$ requires integrating the ODE with the true vector field u_t, which we have established cannot be evaluated pointwise.

S15.3.4 Conditional Drift (Tractable but Uninformative)

One might consider a "conditional" version of drift:

$$\mathcal{E}_c(t) := \mathbb{E}_{q(z), p_t(x|z)} \left\| v_\theta(t, x) - u_t(x|z) \right\|^2. \tag{15}$$

This quantity *is* tractable—it is precisely the CFM training objective evaluated at convergence. However, it measures how well the learned model matches the *conditional* vector fields, not the *marginal* vector field. At any point x, multiple conditional vector fields from different conditions z contribute to the marginal $u_t(x)$ via the weighted average in Eq. (10). Low conditional error does not directly translate to low marginal error without additional regularity assumptions.

S15.4 Fundamental Obstruction: The Marginalization Barrier

The core mathematical obstruction can be summarized as follows:

> *Any quantity that directly measures the discrepancy between the learned generative process and the true generative process requires access to the marginal probability path p_t or marginal vector field u_t. These quantities are defined as expectations over the data distribution (Eqs. 9 and 10), and their evaluation requires intractable marginalization over the conditioning variable z.*

This is not merely a computational inconvenience—it is the fundamental reason why the CFM framework exists. If p_t and u_t were tractable, one could train by directly minimizing $\mathcal{L}_{\mathrm{FM}}(\theta) = \mathbb{E}_{t,p_t}\|v_\theta - u_t\|^2$ rather than the conditional surrogate $\mathcal{L}_{\mathrm{CFM}}(\theta)$.

S15.5 What Can Be Measured: Proxy Metrics

While theoretically principled drift metrics are intractable, practical evaluation of generative models relies on *proxy metrics* that measure properties of the terminal distribution p_1^θ (the distribution of generated samples) against known properties of the target distribution p_1. Examples include:

- **Sample quality metrics**: For molecules, metrics such as chemical validity, PoseBusters validity, and relaxation energy measure whether generated samples satisfy known constraints of the target domain.
- **Distributional statistics**: Comparing marginal distributions of specific features (e.g., functional group frequencies, ring system distributions) between generated and training data.
- **Density estimation proxies**: Negative log-likelihood under auxiliary models, or reconstruction-based metrics.

These metrics implicitly capture accumulated drift by measuring its *effect* at the terminal time $t = 1$, even though they cannot decompose this into contributions along the trajectory. The suite of evaluation metrics we employ (PB-validity, functional group deviation, OOD ring rate, relaxation energy, etc.) represents the current best practice for assessing whether the learned generative process successfully approximates the target distribution.

S15.6 Connection to Self-Conditioning and Distortion

The augmentations we propose in FlowMol3—self-conditioning and coordinate distortion—can be understood as methods that indirectly reduce the effects of distribution drift:

- **Self-conditioning** provides the model with information about its own predicted endpoint during training. This can be viewed as a form of error correction that helps the model remain on valid trajectories even when the marginal velocity approximation is imperfect.
- **Coordinate distortion** augments the training distribution by perturbing molecular geometries. This increases the model's robustness to the compounding of small errors during integration.

While we cannot directly measure how these interventions reduce drift in the theoretical sense, their positive effect on terminal sample quality (as measured by our evaluation metrics) provides indirect evidence of their utility.

S15.7 Conclusion

We have demonstrated that any mathematically rigorous definition of "distribution drift" in learned flow matching models requires access to either the marginal probability path p_t or the marginal vector field u_t. Both quantities are defined as intractable integrals over the data distribution, which is the fundamental computational barrier that the conditional flow matching framework is designed to circumvent.

Consequently, drift cannot be quantified directly. Instead, practical evaluation must rely on proxy metrics that measure properties of terminal samples against known characteristics of the target distribution. The evaluation metrics we report throughout this work (PoseBusters validity, functional group deviation, OOD ring rate, relaxation energy, etc.) represent this indirect assessment of accumulated drift and constitute the standard approach in the field for evaluating generative models that sample from complex, high-dimensional distributions.

References

[1] M. Buttenschoen, G. M. Morris and C. M. Deane, *Chemical Science*, 2024, **15**, 3130–3139.

[2] M. S. Albergo and E. Vanden-Eijnden, *Building Normalizing Flows with Stochastic Interpolants*, 2023, http://arxiv.org/abs/2209.15571, arXiv:2209.15571 [cs, stat].

[3] A. Tong, N. Malkin, G. Huguet, Y. Zhang, J. Rector-Brooks, K. Fatras, G. Wolf and Y. Bengio, *Improving and generalizing flow-based generative models with minibatch optimal transport*, 2023, http://arxiv.org/abs/2302.00482, arXiv:2302.00482 [cs].

[4] I. Gat, T. Remez, N. Shaul, F. Kreuk, R. T. Q. Chen, G. Synnaeve, Y. Adi and Y. Lipman, *Discrete Flow Matching*, 2024, http://arxiv.org/abs/2407.15595, arXiv:2407.15595 [cs].

[5] X. Liu, C. Gong and Q. Liu, *Flow Straight and Fast: Learning to Generate and Transfer Data with Rectified Flow*, 2022, http://arxiv.org/abs/2209.03003, arXiv:2209.03003 [cs].

[6] L. Klein, A. Krämer and F. Noé, *Equivariant flow matching*, 2023, http://arxiv.org/abs/2306.15030, arXiv:2306.15030 [physics, stat].

[7] A. Campbell, J. Yim, R. Barzilay, T. Rainforth and T. Jaakkola, *Generative Flows on Discrete State-Spaces: Enabling Multimodal Flows with Applications to Protein Co-Design*, 2024, http://arxiv.org/abs/2402.04997, arXiv:2402.04997 [cs, q-bio, stat].

[8] B. Jing, S. Eismann, P. N. Soni and R. O. Dror, *Equivariant Graph Neural Networks for 3D Macromolecular Structure*, 2021, http://arxiv.org/abs/2106.03843, arXiv:2106.03843 [cs, q-bio].

[9] A. Schneuing, Y. Du, C. Harris, A. Jamasb, I. Igashov, W. Du, T. Blundell, P. Lió, C. Gomes, M. Welling, M. Bronstein and B. Correia, *Structure-based Drug Design with Equivariant Diffusion Models*, 2023, http://arxiv.org/abs/2210.13695, arXiv:2210.13695 [cs, q-bio].

[10] T. Chen, R. Zhang and G. Hinton, *Analog Bits: Generating Discrete Data using Diffusion Models with Self-Conditioning*, 2023, http://arxiv.org/abs/2208.04202, arXiv:2208.04202 [cs].
